# Travel Time and Distance and Participation in Precision Oncology Trials at the National Cancer Center Hospital

**DOI:** 10.1001/jamanetworkopen.2023.33188

**Published:** 2023-09-15

**Authors:** Yuji Uehara, Takafumi Koyama, Yuki Katsuya, Jun Sato, Kazuki Sudo, Shunsuke Kondo, Tatsuya Yoshida, Hirokazu Shoji, Tatsunori Shimoi, Kan Yonemori, Noboru Yamamoto

**Affiliations:** 1 Department of Experimental Therapeutics, National Cancer Center Hospital, Tokyo, Japan; 2 Department of Thoracic Oncology and Respiratory Medicine, Tokyo Metropolitan Cancer and Infectious Diseases Center, Komagome Hospital, Tokyo, Japan; 3 Department of Precision Cancer Medicine, Center for Innovative Cancer Treatment, Tokyo Medical and Dental University, Tokyo, Japan; 4 Department of Medical Oncology, National Cancer Center Hospital, Tokyo, Japan; 5 Department of Hepatobiliary and Pancreatic Oncology, National Cancer Center Hospital, Tokyo, Japan; 6 Department of Thoracic Oncology, National Cancer Center Hospital, Tokyo, Japan; 7 Department of Gastrointestinal Medical Oncology, National Cancer Center Hospital, Tokyo, Japan

## Abstract

**Importance:**

Genotype-matched trials, which are becoming increasingly important in the precision oncology era, require referrals from institutions providing comprehensive genomic profiling (CGP) testing to those conducting these trials, and the travel burden for trial participation is significant. However, it remains unknown whether travel time or distance are associated with genotype-matched trial participation.

**Objective:**

To assess whether travel time or distance are associated with disparities in genotype-matched trial participation following CGP testing.

**Design, Setting, and Participants:**

This retrospective cohort study from June 2020 to June 2022 included patients with advanced or metastatic solid tumors referred to the National Cancer Center Hospital for participation in genotype-matched trials following CGP testing and discussion by molecular tumor boards. Data were analyzed from June to October 2022.

**Exposures:**

Travel time and distance.

**Main Outcomes and Measures:**

The primary and secondary outcomes were enrollment in genotype-matched trials and all-cancer clinical trials, respectively.

**Results:**

Of 1127 patients (mean [range] age, 62 [16-85] years; 584 women [52%]; all residents of Japan), 127 (11%) and 241 (21%) were enrolled in genotype-matched trials and all-cancer clinical trials, respectively. The overall median (IQR) travel distance and time were 38 (21-107) km and 55 (35-110) minutes, respectively. On multivariable regression with 23 covariates, travel distance (≥100 km vs <100 km) was not associated with the likelihood of genotype-matched trial participation (26 of 310 patients [8%] vs 101 of 807 patients [12%]; odds ratio [OR], 0.64; 95% CI, 0.40-1.02), whereas in patients with travel time of 120 minutes or more, the likelihood of genotype-matched trial participation was significantly lower than those with travel time less than 120 minutes (19 of 276 patients [7%] vs 108 of 851 patients [13%]; OR, 0.51; 95% CI, 0.29-0.84). The likelihood of genotype-matched trial participation decreased as travel time increased from less than 40 (38 of 283 patients [13%]) to 40 to 120 (70 of 568 patients [12%]) and 120 or more (19 of 276 patients [7%]) minutes (OR, 0.74; 95% CI, 0.48-1.17; OR, 0.41; 95% CI, 0.22-0.74, respectively). Neither travel time nor distance were associated with the likelihood of all-cancer clinical trial participation.

**Conclusions and Relevance:**

In this cohort study of patients undergoing CGP testing, an increased travel time was associated with a decreased likelihood of genotype-matched trial participation. This warrants further research on interventions, such as decentralization of clinical trials to mitigate travel burden.

## Key Points

**Question:**

Is there an association between travel time or distance and genotype-matched trial participation among patients referred to the National Cancer Center Hospital following comprehensive genomic profiling?

**Findings:**

In this cohort study of 1227 patients with cancer, travel time (≥120 minutes vs <120 minutes) was associated with participation in a genotype-matched trial; however, travel distance (≥100 km vs <100 km) was not.

**Meaning:**

In this study, an increased travel time was associated with a decreased likelihood of genotype-matched trial participation, suggesting that regional disparities are associated with inequities in precision oncology, which could potentially be addressed with remote technology.

## Teaser

This cohort study evaluates the association between travel time and distance and trial participation among patients referred to the National Cancer Center Hospital in Tokyo, Japan.

## Introduction

The proliferation of next-generation sequencing (NGS) technologies and development of cancer therapeutics targeting genomically defined patient populations have augmented the use of comprehensive genomic profiling (CGP) testing in routine cancer care, particularly for directing patients toward genotype-matched therapies. Approximately 10% of patients who undergo CGP testing receive genotype-matched therapies,^1,2,3,4,5,6,7^ although some researchers have reported a higher proportion.^8,9,10^ Clinical trials are essential for developing and evaluating new genotype-matched therapies. They allow researchers to test new therapies in a controlled setting and collect data on safety and efficacy. Participating in clinical trials can help patients with cancer gain access to new and potentially life-saving therapies that may otherwise be unavailable. The number of genes amenable to genotype-matched trials has been steadily increasing over the past 2 decades,^11^ and the response rate in phase 1 trials has surged by nearly 2-fold to 15% to 25% without an increase in treatment-related death, which has therapeutic implications.^11,12,13,14,15^ Therefore, an inequity in accessibility to these trials suggests an ethical problem.^12^

Several factors can reduce the likelihood of participation in cancer clinical trials, including eligibility and socioeconomic status.^16,17^ The burdens of time and costs associated with travel to the trial sites also account for the low likelihood of cancer clinical trial participation.^18,19,20^ Cancer clinical trials for novel therapies are predominantly offered at a limited number of high-volume facilities and academic centers located in major urban areas.^21^ Patients living in rural areas require referral to high-volume facilities and academic centers for cancer clinical trial participation, which entails travel time, travel expenses, and other costs not covered by insurance (hotel, meals, toll tax, and parking).^7,16,18,22^ In a previous survey of patients with cancer, only 37% were willing to travel to participate in a clinical trial.^23^ Recent research on disparities based on geography has focused on access to standard therapies and cancer clinical trials.^18,24^ However, in the era of precision oncology, the outcomes of regional disparities on participation in genotype-matched trials following CGP testing remains unknown.

The purpose of the current research was to examine the association between travel time and distance and participation in genotype-matched trials following CGP testing and referral to the National Cancer Center Hospital (NCCH) in Tokyo, Japan. We hypothesized that patients who dwelled far away from the NCCH would be less likely to participate in genotype-matched trials. Exploring the association between travel burden and genotype-matched trial participation could inform future efforts to ensure that all patients have access to precision oncology.

## Methods

### Study Design

We conducted a review of 1127 patients referred to the NCCH by treating physicians from external institutions following CGP testing between June 2020 and June 2022. All patients were primarily referred to the NCCH for participation in genotype-matched trials. Most patients had advanced or metastatic solid tumors and had either completed or would soon complete standard therapies, with a few exceptions for patients who had no standard therapy options. Clinical information was obtained from electronic medical records and prospective databases, and data acquisition was locked on October 30, 2022. This research was approved by the NCCH institutional review board. The requirement of informed consent was waived because the data were deidentified. The study is reported in accordance with the Strengthening the Reporting of Observational Studies in Epidemiology (STROBE) reporting guideline.

### Data Collection

The following patient data were collected: demographics, medical history, molecular profiling results, laboratory tests around the time of the initial visit, eligibility of the patient to participate in the cancer clinical trial at the initial visit, history of participation in genotype-matched trials or all-cancer clinical trials, and reasons for participation or nonparticipation in clinical trials. Nonparticipation in clinical trials was often due to multiple factors, and the primary reason for nonparticipation was determined according to the most significant clinical factor for trial participation.

### Molecular Analysis

CGP testing was performed using hybrid capture-based targeted DNA sequencing with FoundationOne CDx (Foundation Medicine), the OncoGuide NCC Oncopanel System (Sysmex Corporation), in-house NGS testing, or in blood-derived cell-free DNA with FoundationOne Liquid CDx (Foundation Medicine) or Guardant360 CDx (Guardant Health).^4,25,26,27^ All patients were referred to the NCCH following discussion by regional molecular tumor boards (MTB) comprising medical oncologists, pediatric oncologists, pathologists, bioinformaticians, genome researchers, and genetic counselors.^3,28^ In addition to reports from the inspection companies, the MTBs used reports containing clinical annotation and information regarding genotype-matched clinical trials from the Center for Cancer Genomics and Advanced Therapeutics national database.^29,30^

### Outcomes

The primary outcome of this research was whether the patients participated in genotype-matched trials, which were defined as those in which patients participated according to CGP results. The specific genomic alterations and corresponding therapies administered in these genotype-matched trials are listed in eTable 1 in Supplement 1. The secondary outcome was whether the patients participated in all-cancer clinical trials (genotype-matched and genotype-nonmatched trials).

### Primary Independent Variables

Travel time was optimally calculated using the Google Maps application from the patient’s residence to the NCCH at 12:00 am on a Monday. Travel distance was calculated using the Google Earth application from the patient’s residence to the NCCH. The data on travel time and distance were dichotomized as less than 120 minutes vs 120 minutes or more and less than 100 km vs 100 or more km, respectively, according to previous research.^24,31,32,33,34^

### Covariates

A total of 23 covariates were preselected according to previous research or expert consensus as follows: age, gender, Eastern Cooperative Oncology Group (ECOG) performance status (PS), body mass index (BMI), tumor type (common vs noncommon cancers), number of lines of prior therapies, number of metastatic sites, liver metastases, brain metastases, pleural effusion or ascites, biopsiability (whether the tumor was biopsiable), neutrophils, hemoglobin, platelets, albumin, creatinine, total bilirubin, lactate dehydrogenase (LDH), aspartate aminotransferase (AST), C-reactive protein (CRP), place of residence (suburban or rural vs urban), type of referring hospital (community hospital vs university hospital or cancer center), and household income.^7,21,23^ Common cancers refer to the top 10 most frequent cancer types in terms of mortality as reported by the World Health Organization, including lung, colorectal, hepatocellular, gastric, breast, esophageal, pancreatic, prostate, cervical, and endometrial cancers.^35^ Biopsiable lesions included lung metastases, hepatic metastases, pelvic tumors, lymph nodes, and those with superficial involvement of the skin, head, neck, or chest wall that were accessible percutaneously through computed tomography guidance, bronchoscopy, or endoscopy. All imaging studies were reviewed by medical oncologists and diagnostic or interventional radiologists to determine the accessibility of the lesion. Furthermore, urban areas were defined as those having a population of 1 million in the city, including Sapporo, Sendai, Saitama, Tokyo, Yokohama, Kawasaki, Chiba, Nagoya, Kyoto, Osaka, Kobe, Hiroshima, and Fukuoka. The median income was obtained from the 2020 census data according to zip code.^36^

### Statistical Analysis

Descriptive statistics were used to summarize baseline characteristics, with the χ^2^ and Wilcoxon rank-sum tests applied to categorical and continuous variables, respectively. Numeric covariates were dichotomized according to standard reference values, while those without standard reference values were dichotomized in accordance with previous research or expert consensus.^3,7,37,38^ For variables with missing data (BMI, neutrophils, hemoglobin, platelets, albumin, LDH, creatinine, total bilirubin, AST, and CRP), a multiple imputation was implemented under the missing at random assumption.^39^

Multivariable logistic regression was estimated to assess the association of travel distance and time with genotype-matched trial participation. All independent variables associated with enrollment in genotype-matched trials (*P* < .20) were included in the multivariable model. The variance inflation factor was used to detect collinearity in the regression model.

In the sensitivity analysis, travel time and distance were trichotomized as follows: less than 40 minutes vs 40 to 120 minutes vs 120 or more minutes; less than 20 km vs 20 to 100 km vs 100 or more km. Propensity scores were estimated by regressing treatment assignment (≥120 minutes vs <120 minutes, ≥100 km vs <100 km) on all 23 covariates and applied using the inverse probability of treatment weighting method (IPTW). The postweighting balance in covariates between treatment groups was estimated using the standardized mean difference (SMD) approach, with an imbalance defined as an SMD greater than 0.1. Furthermore, a complete case analysis was conducted among participants with reported known BMI and laboratory values.

All statistical analyses were performed using R, version 4.1.3 (R Project for Statistical Computing), and significance was set at a 2-sided *P* < .05. Data were analyzed from June to October 2022.

## Results

### Patient Characteristics

**Figure 1.**
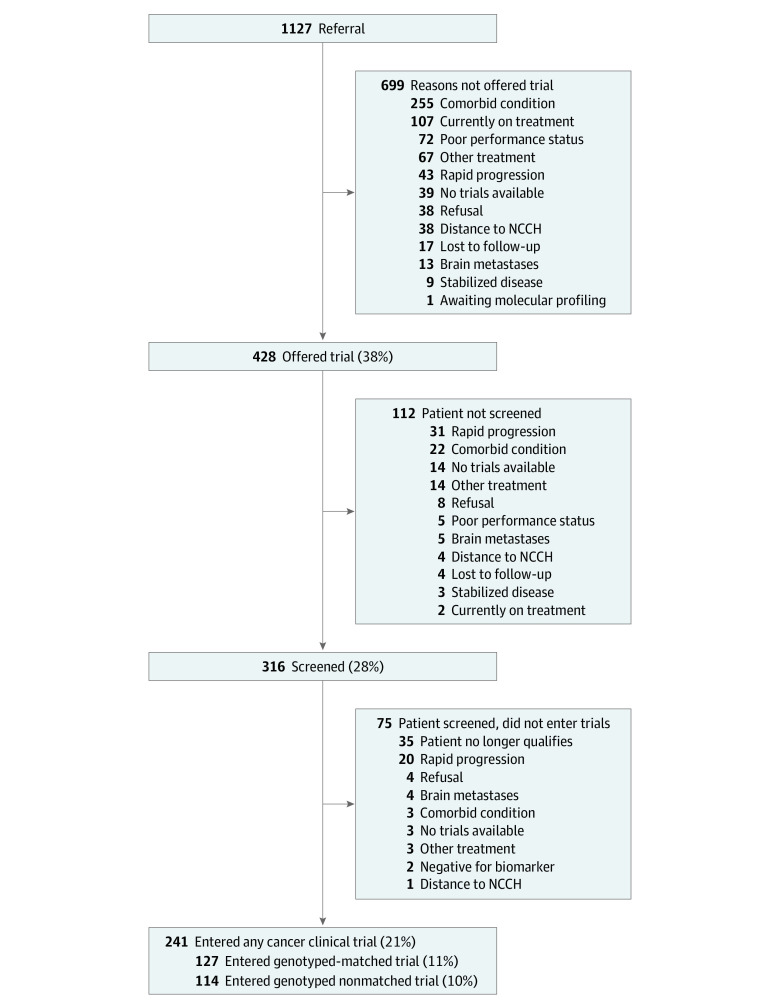
Diagram Illustrating Patient Flow From Referral to Genotype-Matched Trial Participation Abbreviation: NCCH, National Cancer Center Hospital.

Of the 1127 patients (mean [range] age, 62 [16-85] years; 584 women [52%]), all were residents of Japan, and 127 (11%) participated in genotype-matched trials and 241 (21%) participated in all-cancer clinical trials (genotype-nonmatched trials) (Figure 1). The characteristics of the patients included in this research are presented in Table 1 and eTable 2 in Supplement 1. The overall median (IQR) travel time and distance were 55 (35-110) minutes and 38 (21-107) km, respectively. The 127 patients who participated in genotype-matched trials had a median (IQR) travel time and distance of 55 (35-90) minutes and 39 (18-81) km, respectively, and were more likely to have an ECOG-PS of 0, biopsiable lesion, no effusion, higher hemoglobin and albumin levels, lower LDH and CRP levels, and shorter travel time and distance as compared with patients who did not participate in genotype-matched trials. Details regarding the patients who participated in genotype-matched and genotype-nonmatched trials are shown in Table 2 and eTable 3 in Supplement 1. Of the 127 patients who participated in genotype-matched trials, 82 (65%) and 45 (35%) participated in phase 1 and 2 or 3 trials, respectively. Genotype-matched trials were predominantly industry-sponsored (117 of 127 trials [92%]), with a high proportion of targeted monotherapies (87 of 127 trials [69%]).

**Table 1.** Patient Demographics Stratified by Enrollment in Genotype-Matched Trials and All-Cancer Clinical Trials

Demographics	Patients, No, (%)						*P* value
	All patients (N = 1127)	Enrolled in genotype-matched trials (n = 127)	Not enrolled in genotype-matched trials (n = 1000)	*P* value	Enrolled in all-cancer clinical trials (n = 241)	Not enrolled in all-cancer clinical trials (n = 886)	
Age, median (range), y	62 (16-85)	59 (20-80)	62 (16-85)	.01	60 (20-80)	62 (16-85)	.04
Gender							
Men	543 (48)	59 (47)	484 (48)	.75	120 (50)	423 (48)	.62
Women	584 (52)	68 (53)	516 (52)		121 (50)	463 (52)	
Performance status							
0	434 (39)	72 (57)	362 (36)	<.001	146 (61)	288 (33)	<.001
1	607 (54)	54 (43)	553 (55)		94 (39)	513 (58)	
≥2	86 (8)	1 (1)	85 (9)		1 (0.4)	85 (10)	
Body mass index^a^^,^^b^							
<18.5	161 (14)	18 (14)	143 (14)	.99	27 (11)	134 (15)	.02
18.5-25	775 (69)	88 (69)	687 (69)		160 (66)	635 (70)	
≥25	189 (17)	21 (17)	168 (17)		54 (22)	135 (15)	
No. of lines of prior therapies							
Median, (range)	3 (0-12)	3 (0-12)	3 (1-8)	.93	3 (0-12)	3 (0-11)	.03
<2	176 (16)	19 (15)	157 (16)		26 (11)	150 (17)	
≥2	951 (84)	108 (85)	843 (84)		215 (89)	736 (83)	
No. of metastatic sites							
Median, (range)	2 (0-7)	2 (1-5)	2 (0-7)	.26	2 (0-5)	2 (0-7)	.021
<2	354 (31)	46 (36)	308 (31)		91 (38)	263 (30)	
≥2	773 (69)	81 (64)	692 (69)		150 (62)	623 (70)	
Liver metastases							
No	616 (55)	71 (55)	545 (54)	.84	144 (60)	472 (53)	.09
Yes	511 (45)	56 (45)	455 (46)		97 (40)	414 (47)	
Brain metastases							
No	1063 (94)	123 (97)	940 (94)	.27	233 (97)	830 (94)	.10
Yes	64 (6)	4 (3)	60 (6)		8 (3)	56 (6)	
Pleural effusions or ascites							
No	963 (85)	120 (95)	843 (84)	.003	229 (95)	734 (83)	<.001
Yes	164 (15)	7 (5)	157 (16)		12 (5)	152 (17)	
Biopsiable							
No	307 (27)	22 (17)	285 (28)	.01	38 (16)	269 (30)	<.001
Yes	820 (73)	105 (83)	715 (72)		203 (84)	617 (70)	
≥5	111 (10)	3 (2)	108 (11)		3 (1)	108 (12)	
Place of residence							
Suburban/rural	844 (75)	96 (74)	748 (81)	.93	141 (75)	663 (75)	>.99
Urban	283 (25)	31 (24)	252 (25)		60 (25)	223 (25)	
Referring hospital							
Community hospital	585 (52)	61 (48)	524 (52)	.38	119 (49)	466 (53)	.05
University hospital	310 (28)	34 (27)	276 (28)		59 (25)	251 (28)	
Cancer center	232 (21)	32 (25)	200 (20)		63 (26)	169 (19)	
Median income (by zip code)							
<$25 000	124 (11)	12 (10)	112 (11)	.66	26 (11)	98 (11)	>.99
≥$25 000	1003 (89)	115 (90)	888 (89)		215 (89)	788 (89)	
Travel time							
Median (IQR)	55 (35-110)	55 (35-90)	60 (40-112)	.01	39 (23-83)	60 (40-112)	.07
<120 min	851 (76)	108 (85)	743 (74)		196 (81)	655 (74)	
≥120 min	276 (24)	19 (15)	257 (26)		45 (19)	231 (26)	
Travel distance							
Median (IQR)	38 (21-107)	39 (18-81)	38 (21-116)	.03	55 (40-100)	38 (21-116)	.04
<100 km	817 (72)	101 (80)	716 (72)		187 (78)	630 (71)	
≥100 km	310 (28)	26 (20)	284 (28)		54 (22)	256 (29)	

^a^ Body mass index is calculated as weight in kilograms divided by height in meters squared.

^b^ Missing data for body mass index: 2 participants.

**Table 2.** Characteristics of Patients Participating in Genotype-Matched and Genotype-Nonmatched Trials

Characteristics	Patients, No (%)			*P* value
	All-cancer clinical trials (n = 241)	Genotype-matched trials (n = 127)	Genotype-nonmatched trials (n = 114)	
Age, median (range), y	60 (20-80)	58 (20-80)	60 (38-79)	.13
Gender				
Men	120 (50)	59 (47)	61 (54)	.34
Women	121 (50)	68 (53)	53 (46)	
Performance status				
0	146 (61)	72 (57)	74 (65)	.24
≥1	95 (39)	55 (43)	40 (35)	
Body mass index^a^				
<18.5	27 (11)	18 (14)	9 (8)	.04
18.5-25	160 (66)	88 (69)	72 (63)	
≥25	54 (22)	21 (17)	33 (29)	
No. of lines of prior therapies				
Median, (range)	3 (0-12)	3 (1-8)	4 (0-12)	.046
<2	26 (11)	19 (15)	7 (6)	
≥2	215 (89)	108 (85)	107 (94)	
No. of metastatic sites				
Median, (range)	2 (0-5)	2 (1-5)	2 (0-5)	.70
<2	91 (38)	46 (36)	45 (40)	
≥2	150 (62)	81 (64)	69 (60)	
Liver metastases				
No	144 (60)	71 (55)	73 (64)	.25
Yes	97 (40)	56 (45)	41 (36)	
Brain metastases				
No	233 (97)	123 (97)	110 (96)	>.99
Yes	8 (3)	4 (3)	4 (4)	
Pleural effusions or ascites				
No	229 (95)	120 (95)	109 (96)	.92
Yes	12 (5)	7 (5)	5 (4)	
Biopsiable				
No	38 (16)	22 (17)	16 (14)	.60
Yes	203 (84)	105 (83)	98 (86)	
Place of residence				
Suburban/rural	141 (75)	96 (74)	85 (75)	.97
Urban	60 (25)	31 (24)	29 (25)	
Referring hospital				
Community hospital	119 (49)	61 (48)	58 (51)	.68
University hospital	59 (25)	34 (27)	25 (22)	
Cancer center	63 (26)	32 (25)	31 (27)	
Median income (by zip code)				
<$25 000	26 (11)	12 (10)	14 (12)	.62
≥$25 000	215 (89)	115 (90)	100 (88)	
Trial phase				
Phase 1	184 (77)	82 (65)	102 (90)	<.001
Phase 2	48 (20)	42 (33)	6 (5)	
Phase 3	9 (4)	3 (2)	6 (5)	
Sponsor type				
Industry	231 (96)	117 (92)	114 (100)	.01
Principal investigator	10 (4)	10 (8)	0	
Investigational agents				
Targeted monotherapy	137 (57)	87 (69)	50 (44)	<.001
Targeted drug combination	14 (6)	12 (9)	2 (2)	
Targeted drug and chemotherapy	2 (1)	1 (1)	1 (1)	
Immunotherapy	65 (27)	12 (9)	53 (47)	
Targeted drug and immunotherapy	23 (10)	15 (12)	8 (7)	
Travel time				
Median (IQR)	39 (23-83)	39 (18-81)	40 (24-97)	.17
<120 min	196 (81)	108 (85)	88 (77)	
≥120 min	45 (19)	19 (15)	26 (23)	
Travel distance				
Median (IQR)	55 (40-100)	55 (35-90)	55 (40-100)	.07
<100 km	187 (78)	101 (80)	86 (75)	
≥100 km	54 (22)	26 (20)	28 (25)	

^a^ Body mass index is calculated as weight in kilograms divided by height in meters squared.

### Association Between Travel Time and Genotype-Matched Trial Participation

**Figure 2.**
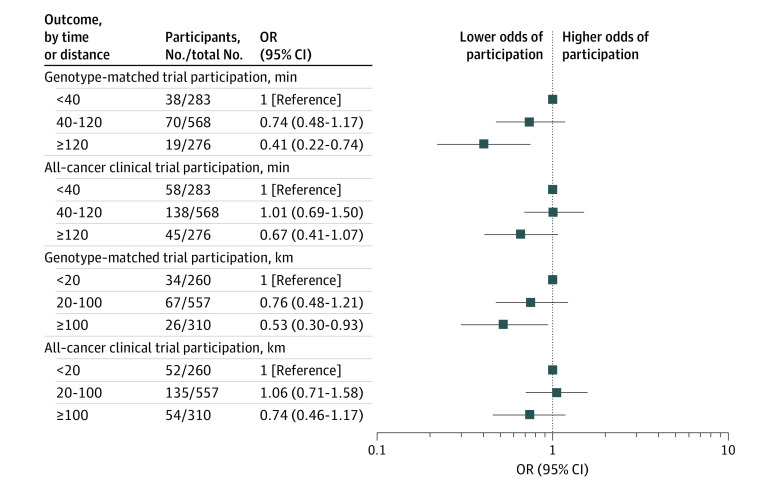
Genotype-Matched and All-Cancer Clinical Trial Participation by Travel Time and Distance Abbreviation: OR, odds ratio.

After adjusting for covariates, patients with a travel time of 120 or more minutes were found to be significantly less likely to participate in genotype-matched trials than those with a travel time of less than 120 minutes (19 of 276 patients [7%] vs 108 of 851 patients [13%]; odds ratio [OR], 0.51; 95% CI, 0.29-0.84; *P* = .01) (Table 3). In addition, the likelihood of genotype-matched trial participation decreased as travel time increased from less than 40 (38 of 283 patients [13%]) to 40 to 120 (70 of 568 patients [12%]) and 120 or more minutes (19 of 276 patients [7%]) (OR, 0.74; 95% CI, 0.48-1.17; OR, 0.41; 95% CI, 0.22-0.74, respectively) (Figure 2).

**Table 3.** Univariable and Multivariable Analyses of Factors Associated With Genotype-Matched Trial Participation by Travel Time or Distance to the National Cancer Center Hospital

Variable	Univariable analysis		Multivariable analysis^a^	
	OR (95% CI)	*P* value	OR (95% CI)	*P* value
Travel time				
Travel time ≥120 min (vs <120 min)	0.55 (0.32-0.88)	.02	0.51 (0.29-0.84)	.01
Age ≥60 y (vs <60 y)	0.69 (0.47-0.99)	.047	0.63 (0.43-0.93)	.02
Gender, women (vs men)	1.04 (0.72-1.51)	.82	NA	NA
Performance status, ≥1 (vs 0)	0.45 (0.31-0.65)	<.001	0.58 (0.39-0.85)	.01
Body mass index, <18.5 (vs ≥18.5)^b^	0.87 (0.52-1.40)	.59	NA	NA
Tumor type, noncommon cancers (vs common cancers)	0.98 (0.52-1.72)	.95	NA	NA
No. of lines of prior therapies, ≥2 (vs <2)	1.06 (0.65-1.82)	.83	NA	NA
No. of metastatic sites, ≥2 (vs <2)	0.81 (0.56-1.21)	.30	NA	NA
Liver metastases, yes (vs no)	0.98 (0.67-1.42)	.91	NA	NA
Brain metastases, yes (vs no)	0.51 (0.15-1.26)	.21	NA	NA
Pleural effusions or ascites, yes (vs no)	0.31 (0.13-0.64)	.004	0.37 (0.15-0.76)	.01
Biopsiable, no (vs yes)	0.53 (0.32-0.83)	.01	0.50 (0.30-0.80)	.01
Neutrophils (/μL), <1500 (vs ≥1500)	0.41 (0.10-1.13)	.14	0.41 (0.10-1.16)	.14
Hemoglobin (g/dL), <9 (vs ≥9)	0.25 (0.06-0.67)	.02	0.48 (0.11-1.39)	.24
Platelets ( × 10^3^/μL), <100 (vs ≥100)	0.44 (0.11-1.22)	.17	0.43 (0.10-1.23)	.17
Albumin (g/dL), <3.5 (vs ≥3.5)	0.22 (0.11-0.40)	<.001	0.46 (0.22-0.88)	.03
LDH (U/L), ≥2.5 × ULN (vs <2.5 × ULN)	0.15 (0.02-0.48)	.01	0.24 (0.04-0.83)	.06
Creatinine (mg/dL), ≥1.5 (vs <1.5)	0.52 (0.03-2.60)	.53	NA	NA
Total bilirubin (mg/dL), ≥1.5 × ULN (vs <1.5 × ULN)	0.98 (0.05-5.43)	.99	NA	NA
AST (U/L), ≥3.0 × ULN (vs <3.0 × ULN)	0.25 (0.01-1.17)	.17	0.32 (0.02-1.64)	.28
CRP (mg/dL), ≥5 (vs <5)	0.20 (0.05-0.54)	<.001	0.44 (0.11-1.30)	.19
Place of residence, suburban/rural (vs urban)	1.04 (0.69-1.62)	.85	NA	NA
Referring hospital, community (vs hospital university hospital or cancer center)	0.84 (0.58-1.21)	.35	NA	NA
Median income (by zip code), <$25 000 (vs ≥$25 000)	0.91 (0.48-1.62)	.77	NA	NA
Travel distance				
Travel distance, ≥100 km (vs <100 km)	0.68 (0.43-1.05)	.09	0.64 (0.40-1.02)	.06
Age, ≥60 y (vs <60 y)	0.69 (0.47-0.99)	.047	0.63 (0.43-0.93)	.03
Gender, women (vs men)	1.04 (0.72-1.51)	.82	NA	NA
Performance status, 1 (vs 0)	0.45 (0.31-0.65)	<.001	0.58 (0.39-0.85)	.01
Body mass index, <18.5 (vs ≥18.5)^b^	0.87 (0.52-1.40)	.59	NA	NA
Tumor type, noncommon cancers (vs common cancers)	0.98 (0.52-1.72)	.95	NA	NA
No. of lines of prior therapies, ≥2 (vs <2)	1.06 (0.65-1.82)	.83	NA	NA
No. of metastatic sites, ≥2 (vs <2)	0.81 (0.56-1.21)	.30	NA	NA
Liver metastases, yes (vs no)	0.98 (0.67-1.42)	.91	NA	NA
Brain metastases, yes (vs no)	0.51 (0.15-1.26)	.21	NA	NA
Pleural effusions or ascites, yes (vs no)	0.31 (0.13-0.64)	.004	0.37 (0.15-0.76)	.02
Biopsiable, no (vs yes)	0.53 (0.32-0.83)	.01	0.50 (0.29-0.80)	.01
Neutrophils (/μL), <1500 (vs ≥1500)	0.41 (0.10-1.13)	.14	0.41 (0.10-1.16)	.14
Hemoglobin (g/dL), < 9 (vs ≥9)	0.25 (0.06-0.67)	.02	0.48 (0.11-1.39)	.24
Platelets ( × 10^3^/μL), <100 (vs ≥100)	0.44 (0.11-1.22)	.17	0.43 (0.10-1.23)	.17
Albumin (g/dL), <3.5 (vs ≥3.5)	0.22 (0.11-0.40)	<.001	0.46 (0.22-0.88)	.02
LDH (U/L), ≥2.5 × ULN (vs <2.5 × ULN)	0.15 (0.02-0.48)	.01	0.24 (0.04-0.83)	.06
Creatinine (mg/dL), ≥1.5 (vs <1.5)	0.52 (0.03-2.60)	.53	NA	NA
Total bilirubin (mg/dL), ≥1.5 × ULN (vs <1.5 × ULN)	0.98 (0.05-5.43)	.99	NA	NA
AST (U/L), ≥3.0 × ULN (vs <3.0 × ULN)	0.25 (0.01-1.17)	.17	0.32 (0.02-1.64)	.26
CRP (mg/dL), ≥5 (vs < 5)	0.20 (0.05-0.54)	<.001	0.44 (0.11-1.30)	.19
Place of residence, suburban/rural (vs urban)	1.04 (0.69-1.62)	.85	NA	NA
Referring hospital, community (vs hospital university hospital or cancer center)	0.84 (0.58-1.21)	.35	NA	NA
Median income (by zip code), <$25 000 (vs ≥$25 000)	0.91 (0.48-1.62)	.77	NA	NA

Abbreviations: AST, aspartate transaminase; CRP, C-reactive protein; LDH, lactate dehydrogenase; NA, not applicable; OR, odds ratio; ULN, upper normal limit.

To convert albumin to grams per liter, multiply by 10; creatinine to μmol per liter, multiply by 76.25; CRP to milligrams per liter, multiply by 10; hemoglobin to grams per liter, multiply by 10; neutrophils to ×10^9^ per liter, multiply by 0.001; platelets to ×10^9^ per liter, multiply by 1.0; total bilirubin μmol per liter, multiply by 17.104.

^a^ All variables with *P* < .20 were entered into the full model analysis.

^b^ Body mass index is calculated as weight in kilograms divided by height in meters squared.

### Association Between Travel Distance and Genotype-Matched Trial Participation

After adjusting for covariates, travel distance (≥100 km vs <100 km) was not associated with the likelihood of genotype-matched trial participation (26 of 310 patients [8%] vs 101 of 817 patients [12%]; OR, 0.64; 95% CI, 0.40-1.02; *P* = .06) (Table 3). However, the likelihood of genotype-matched trial participation decreased as travel distance increased from less than 20 (34 of 260 patients [13%]) to 20 to 100 (67 of 557 patients [12%]) and 100 or more (26 of 310 patients [8%]) km (OR, 0.76; 95% CI, 0.48-1.21; OR, 0.53; 95% CI, 0.30-0.93) (Figure 2).

### Association Between Travel Time and Distance and All-Cancer Clinical Trial Participation

After adjusting for covariates, travel time (≥120 minutes vs <120 minutes) was not associated with the likelihood of participation in all-cancer clinical trials (45 of 276 [16%] vs 196 of 851 patients [23%]; OR, 0.70; 95% CI, 0.47-1.04; *P* = .08) (eTable 4 in Supplement 1). Travel distance (≥100 km vs <100 km) was also not associated with the likelihood of participation in all-cancer clinical trials (54 of 310 [17%] vs 187 of 817 patients [23%], respectively; OR, 0.76; 95% CI, 0.51-1.10; *P* = .15) (eTable 5 in Supplement 1).

### Correlation Between Travel Time and Travel Distance

The areas of difference between travel distance less than 100 km and travel time less than 120 minutes are shown in eFigure 1 in Supplement 1. Travel time and distance were significantly correlated among overall patients and patients participating in genotype-matched trials (Spearman correlation coefficient, 0.98; *P* < .001; and 0.98; *P* < .001, respectively) (eFigure 2 in Supplement 1); whereas, of 35 patients with a travel distance of 100 or more km but travel time of less than 120 minutes, 7 (20%) participated in genotype-matched trials (eFigure 3 in Supplement 1), which was higher than the overall genotype-matched trial participation rate (11%).

### Sensitivity Analyses

We also performed propensity score adjustment using the IPTW method. Patients with a travel time of 120 or more minutes were significantly less likely to participate in genotype-matched trials compared with those with a travel time of less than 120 minutes (OR, 0.48; 95% CI, 0.28-0.81; *P* = .01). Patients with a travel distance of 100 or more km were also significantly less likely to participate in genotype-matched trials compared with those with a travel distance less than 100 km (OR, 0.55; 95% CI, 0.33-0.91; *P* = .02). The odds ratios for the complete-case analyses were consistent with those of the primary analysis.

## Discussion

In this research, we found that an increased travel time (≥120 minutes vs <120 minutes) was significantly associated with a decreased likelihood of genotype-matched trial participation, even after adjusting for covariates or IPTW analysis. However, longer travel distances had a modest association with a lower likelihood of participating in a genotype-matched trial. These results suggest the presence of regional inequities in genotype-matched trial participation and underscore the need for targeted interventions to improve access for patients who reside far from trial sites.

Travel burden could potentially be one of the biggest barriers to patient participation in cancer clinical trials.^20,23,40^ Travel expenses are not typically covered by health care payers; therefore, patients encounter significant out-of-pocket expenditure for visits stipulated in the study protocol for clinical trials. However, recent research has not shown a clear association between travel distance and participation in cancer clinical trials.^3,7^ Consistent with previous research, our findings showed that travel distance (≥100 km vs <100 km) was not significantly associated with the likelihood of genotype-matched trial participation, although we did find an association between a longer travel distance (when separated into 3 distance categories of ≥100 km, 20-100 km, and <20 km) and a lower likelihood of genotype-matched trial participation. In contrast, travel time (≥120 minutes vs <120 minutes) was significantly associated with the likelihood of genotype-matched trial participation. Therefore, when assessing regional disparities in precision oncology, travel time may be preferable over travel distance as an indicator of travel burden.

Our findings revealed that travel burden (time and distance) was not associated with the likelihood of participation in all-cancer clinical trials, unlike in genotype-matched trials. Genotype-matched trials are more attractive to patients in terms of antitumor efficacy than genotype-nonmatched trials.^14^ However, the slots available in genotype-matched trials tend to fill up faster than those in genotype-nonmatched trials, and quick decision-making between patients and clinical trial investigators may be more critical in genotype-matched trial participation. Travel burden can be a financial and psychological barrier to patients when deciding whether to participate in cancer clinical trials, leading to delays in consent and potentially causing them to miss opportunities to enroll in genotype-matched trials.^23^ Additionally, the majority of genotype-matched trials require patients to arrive at the hospital early in the morning because the study protocols require that oral drugs, such as tyrosine kinase inhibitors, be administered in the morning. It is imperative to ensure that patients living more than 120 minutes away from large cancer centers have access to genotype-matched trials.

Regional disparities in genotype-matched trial participation remain poorly addressed. Limiting regional disparities in precision oncology is crucial to ensure fair access to promising genotype-matched trials. Although numerous pharmaceutical companies, through industry-sponsored trials, endeavor to mitigate out-of-pocket expenses through a financial assistance program, such aid may be insufficient for patients living further away from trial sites. Providing additional support for out-of-pocket expenses for travel to trial sites may help reduce disparity in genotype-matched trial enrollment.^41^ Moreover, the physical burden of long travel can be arduous for patients with advanced cancer. The greater adoption of decentralization tools for locations with a travel time of 120 or more minutes could alleviate such a disparity. In a previous cross-sectional survey for patients with cancer,^32^ most patients (60%-85%) responded that they would be more likely to participate in a clinical trial that required travel if the travel burden could be mitigated via remote technology. Some decentralized, patient-centric trials are currently ongoing. These include the TCF-001 TRACK,^42^ which involves obtaining remote consent to ensure study access regardless of geographic location, and Alpha-T,^43^ which allows for most assessments to be conducted at home. We are planning to conduct decentralized clinical trials to enhance genotype-matched trial participation by minimizing regional disparities. Furthermore, our findings may inform strategies for industries to increase accrual for study trials by mitigating the travel burden as a participation barrier.

### Strengths and Limitations

To our knowledge, this research is the first to investigate the association between travel burden and participation in genotype-matched trials among patients referred to trial sites after CGP testing. Prior research regarding travel burden has focused on participation in all-cancer clinical trials after small NGS panels and limited molecular testing, and did not reflect modern precision oncology.^7^ In addition, this research is the first to use the Google Maps calculator to measure the optimal travel time for patients in cancer clinical trials. Furthermore, the strengths of this research include the consideration of a broad range of clinical characteristics and diverse tumor types, referral to the largest national cancer center in the country, and a control group of patients who did not participate in genotype-matched trials due to travel or other perceived burdens.

This research had several limitations. First, although this research was conducted at the largest cancer center in Japan with comparable rates of participation in genotype-matched trials with those observed in other countries, it was a single-site study, and the results may not be generalizable to patients from other countries.^1,3^ Second, there were no objective criteria for genotype-matched or genotype-nonmatched trials and criteria were based on the scientific judgment of multiple medical oncologists. Third, selection bias may limit the generalizability of this research due to its retrospective design. Patients living far from the NCCH in this research may generally have been more willing to participate in cancer clinical trials. Some patients may have been enrolled in clinical trials conducted at other institutions. An additional limitation is that measures of marital status and family functioning were not included, which could have potentially influenced genotype-matched trial participation. Instead of individual socioeconomic status, income was extrapolated from zip code–level census data.

## Conclusions

In this cohort study, an increased travel time was associated with a decreased likelihood of genotype-matched trial participation after CGP testing and referral to the NCCH. Genotype-matched trials have become increasingly important as the newest available therapy in the precision oncology era; therefore, regional disparities in genotype-matched trial participation underscore the potential need for interventions addressing inequities, such as the decentralization of clinical trials to mitigate travel burden.

## Notes

Supplement 1. **eTable 1. **Genomic Alterations and Corresponding Therapies in Genotype-Matched Trials **eTable 2. **Characteristics of Patients Stratified by Enrollment in Genotype-Matched Trials or All-Cancer Clinical Trials **eTable 3. **Characteristics of Patients Participating in Genotype-Matched and Genotype-Nonmatched Trials **eTable 4. **Univariable and Multivariable Analyses of Factors Associated With All-Cancer Clinical Trial Participation by Travel Time to the Hospital **eTable 5. **Univariable and Multivariable Analyses of Factors Associated With All-Cancer Clinical Trial Participation by Travel Distance to the Hospital **eFigure 1. **Travel Time and Distance Map Centered on the National Cancer Center Hospital **eFigure 2. **Correlation Between Travel Time and Distance **eFigure 3. **Scatter Plot of Patients With a Travel Time Less Than 120 Minutes and Travel Distance 100 or More km

Supplement 2.
Data Sharing Statement
