# Supplementary material for: Travel Time and Distance and Participation in Precision Oncology Trials at the National Cancer Center Hospital
Source: JAMA Netw Open. 2023 Sep 15;6(9):e2333188. doi: 10.1001/jamanetworkopen.2023.33188 (PMC10504617; doi:10.1001/jamanetworkopen.2023.33188)
Supplement: Supplement 1. — eTable 1. Genomic Alterations and Corresponding Therapies in Genotype-Matched Trials eTable 2. Characteristics of Patients Stratified by Enrollment in Genotype-Matched Trials or All-Cancer Clinical Trials eTable 3. Characteristics of Patients Participating in Genotype-Matched and Genotype-Nonmatched Trials eTable 4. Univariable and Multivariable Analyses of Factors Associated With All-Cancer Clinical Trial Participation by Travel Time to the Hospital eTable 5. Univariable and Multivariable Analyses of Factors Associated With All-Cancer Clinical Trial Participation by Travel Distance to the Hospital eFigure 1. Travel Time and Distance Map Centered on the National Cancer Center Hospital eFigure 2. Correlation Between Travel Time and Distance eFigure 3. Scatter Plot of Patients With a Travel Time Less Than 120 Minutes and Travel Distance 100 or More km [file jamanetwopen-e2333188-s001.pdf]

Supplemental Online Content

Uehara Y, Koyama T, Katsuya Y, et al. Travel Time and Distance and Participation in Oncology Trials at a National Cancer Center. *JAMA Netw Open*. 2023;6(9):e2333188. doi:10.1001/jamanetworkopen.2023.33188

eTable 1. Genomic Alterations and Corresponding Therapies in Genotype-Matched Trials

eTable 2. Characteristics of Patients Stratified by Enrollment in Genotype-Matched Trials or All–Cancer Clinical Trials

eTable 3. Characteristics of Patients Participating in Genotype-Matched and Genotype-Nonmatched Trials

eTable 4. Univariable and Multivariable Analyses of Factors Associated With All–Cancer Clinical Trial Participation by Travel Time to the Hospital

eTable 5. Univariable and Multivariable Analyses of Factors Associated With All–Cancer Clinical Trial Participation by Travel Distance to the Hospital

eFigure 1. Travel Time and Distance Map Centered on the National Cancer Center Hospital

eFigure 2. Correlation Between Travel Time and Distance

eFigure 3. Scatter Plot of Patients With a Travel Time Less Than 120 Minutes and Travel Distance 100 or More km

This supplemental material has been provided by the authors to give readers additional information about their work.

© 2023 Uehara Y et al. *JAMA Network Open.*

eTable 1. Genomic Alterations and Corresponding Therapies in Genotype-Matched Trials

**Genomic alterations**	**Corresponding therapy**
KRAS G12C	KRAS-G12C inhibitor
KRAS G12S	SHP2 inhibitor
KRAS G12D	KRAS inhibitor
KRAS G12D	MEK inhibitor
KRAS G12D	SHP2 inhibitor
KRAS G12D	MAPK pathway inhibitor
KRAS G12R	KRAS inhibitor
KRAS G12V	MEK inhibitor
KRAS A146T	MAPK pathway inhibitor
EGFR exon 19del	ERBB inhibitor
BRCA1 splice site 5277+1G>C	PARP inhibitor
BRCA2 R18fs*12	PARP inhibitor
BRCA2 E826	PARP inhibitor
BRCA2 K1440fs*4	PARP inhibitor
BRCA2 I1859fs*3	PARP inhibitor
BRCA2 R2318*	PARP inhibitor
BRCA2 splice site 9117G>A	PARP inhibitor
BRAF V600E	MEK inhibitor, BRAF inhibitor
BRAF V600E	MEK inhibitor, BRAF inhibitor, EGFR inhibitor
BRAF N486_P490del	MEK inhibitor, BRAF inhibitor
BRAF N486_P490 del	ERK1/2 inhibitor
HER2 amplification	HER2 inhibitor
HER2 S310F	HER2 inhibitor
HER2 S310Y	HER2 inhibitor
HER2 R678Q	HER2 inhibitor
HER2 T733I	HER2 inhibitor
HER2 A775_G776insYVMA	HER2 inhibitor
MDM2 amplification	MDM2 inhibitor
TP53 C238G	WEE1 inhibitor
KIF5B-RET fusion	RET inhibitor
APC Q236*	Wnt/β-catenin signaling inhibitor
APC K516N	Wnt/β-catenin signaling inhibitor
APC splice site 835-8A>G	Wnt/β-catenin signaling inhibitor
APC R876*	Wnt/β-catenin signaling inhibitor
APC R1051*	Wnt/β-catenin signaling inhibitor
APC E1168*	Wnt/β-catenin signaling inhibitor
APC S1327*	Wnt/β-catenin signaling inhibitor
APC R1450*	Wnt/β-catenin signaling inhibitor
APC Q1367*	Wnt/β-catenin signaling inhibitor
APC F1396fs*20	Wnt/β-catenin signaling inhibitor
APC S1465fs*3	Wnt/β-catenin signaling inhibitor
APC T1556fs*3	Wnt/β-catenin signaling inhibitor
IDH1 R132C	IDH1 inhibitor
ROS1-GOPC fusion	ROS1 inhibitor
ATM R1898Q	ATR inhibitor
ATM W2769*	ATR inhibitor
FGFR1 amplification	FGFR inhibitor
FGFR2-CIT fusion	FGFR inhibitor
FGFR2-YPEL5 fusion	FGFR inhibitor
FGFR2-FOXP1 fusion	FGFR inhibitor
FGFR2 amplification	FGFR inhibitor
FGFR2 H167_N173del	FGFR inhibitor
FGFR2 F276C	FGFR inhibitor

© 2023 Uehara Y et al. *JAMA Network Open.*

**Genomic alterations**	**Corresponding therapy**
FGFR3 S249C	FGFR inhibitor
FGFR3 fusion (unknown fusion partner)	FGFR inhibitor
ALK amplification	ALK inhibitor
RET V804M	RET inhibitor
PIK3CA N345T	PI3K inhibitor
MTAP loss	PRMT5 inhibitor
High TMB[a]	Anti-PD-1/PD-L1 antibody

Abbreviation: TMB, tumor mutational burden.

[a]High TMB was defined as ≥10 mutations per megabase.

© 2023 Uehara Y et al. *JAMA Network Open.*

eTable 2. Characteristics of Patients Stratified by Enrollment in Genotype-Matched Trials or All–Cancer Clinical Trials (tumor Type and Laboratory Values, n = 1,127)

**Demographics**	**All patients (N = 1127)**	**Enrolled in genotype-matched trials (n = 127)**	**Not enrolled in genotype-matched rials (n = 1000)**	***P***	**Enrolled in all-cancer clinical trials (n = 241)**	**Not enrolled in all-cancer clinical trials (n = 886)**	***P***
Tumor type, n (%)							
Gastrointestinal	335 (30)	44 (35)	291 (29)	.36	86 (36)	249 (28)	.07
Hepatobiliary	314 (28)	30 (24)	284 (28)		56 (23)	258 (29)	
Gynecologic	179 (16)	22 (17)	157 (16)		40 (17)	139 (16)	
Breast	61 (5)	4 (3)	57 (6)		9 (4)	52 (6)	
Lung	56 (5)	9 (7)	47 (5)		18 (8)	38 (4)	
Sarcoma	43 (4)	8 (6)	35 (4)		11 (5)	32 (4)	
Genitourinary	42 (4)	4 (3)	38 (4)		10 (4)	32 (4)	
Head and neck	23 (2)	3 (2)	20 (2)		4 (2)	19 (2)	
Skin, melanoma	14 (1)	0 (0)	14 (1)		1 (0.4)	13 (2)	
Central nervous system	14 (1)	1 (1)	13 (1)		1 (0.4)	13 (2)	
Carcinoma of unknown primary	10 (1)	1 (1)	9 (1)		1 (0.4)	9 (1)	
Other[a]	36 (3)	1 (1)	35 (4)		4 (2)	32 (4)	

© 2023 Uehara Y et al. *JAMA Network Open.*

**Demographics**	**All patients (N = 1127)**	**Enrolled in genotype-matched trials (n = 127)**	**Not enrolled in genotype-matched trials (n = 1000)**	***P***	**Enrolled in all-cancer clinical trials (n = 241)**	**Not enrolled in all-cancer clinical trials (n = 886)**	***P***
Neutrophils (/μL)[b], n (%)							
<1,500	59 (5)	3 (2)	56 (6)	.15	4 (2)	55 (6)	.005
≥1,500	1021 (91)	124 (98)	897 (90)		237 (98)	784 (89)	
Hemoglobin (g/dL)[c], n (%)							
<9	92 (8)	3 (2)	89 (9)	.01	4 (2)	88 (10)	<.001
≥9	988 (88)	124 (98)	864 (86)		237 (98)	751 (85)	
Platelets ($\times 10^3$/μL)[d], n (%)							
<100	55 (5)	3 (2)	52 (5)	.20	4 (2)	51 (6)	.01
≥100	1025 (91)	124 (98)	901 (90)		237 (98)	788 (90)	
Albumin (g/dL)[e], n (%)							
<3.5	257 (23)	11 (9)	246 (25)	<.001	19 (8)	238 (27)	<.001
≥3.5	818 (73)	116 (91)	702 (70)		222 (92)	596 (67)	
LDH (U/dL)[f], n (%)							
<2.5 × ULN	1000 (89)	125 (98)	875 (88)	.02	236 (98)	764 (87)	.01
≥2.5 × ULN	74 (7)	2 (2)	72 (7)		5 (2)	69 (8)	
Creatinine (mg/dL)[g], n (%)							
<1.5	1063 (94)	126 (99)	937 (94)	.76	240 (99)	823 (93)	.21
≥1.5	16 (1)	1 (1)	15 (2)		1 (1)	15 (2)	

© 2023 Uehara Y et al. *JAMA Network Open.*

Demographics	All patients (N = 1127)	Enrolled in genotype-matched trials (n = 127)	Not enrolled in genotype-matched trials (n = 1000)	*P*	Enrolled in all-cancer clinical trials (n = 241)	Not enrolled in all-cancer clinical trials (n = 886)	*P*
Total bilirubin (mg/dL)[h], n (%)							
<1.5 × ULN	1059 (94)	126 (99)	933 (93)	>.99	240 (99)	819 (92)	.67
≥1.5 × ULN	9 (1)	1 (1)	8 (1)		1 (1)	8 (1)	
AST (U/L)[i], n (%)							
<3.0 × ULN	1047 (93)	126 (99)	921 (92)	.21	240 (99)	807 (91)	.02
≥3.0 × ULN	32 (3)	1 (1)	31 (3)		1 (1)	31 (4)	
CRP (mg/dL)[j], n (%)							
<5	931 (83)	123 (97)	808 (81)	.002	235 (98)	696 (79)	<.001
≥5	111 (10)	3 (2)	108 (11)		3 (1)	108 (12)	

[a] Other: neuroendocrine tumor (13), thyroid cancer (8), germ cell tumor (4), thymic cancer (2), mesothelioma (2), adrenal cortical cancer (2), malignant peripheral nerve sheath tumor (1), desmoid tumor (1), Bowen's disease (1), epithelioid hemangioendothelioma (1), and penile cancer (1).
[b] Missing data for neutrophils: n = 47.
[c] Missing data for hemoglobin: n = 47.
[d] Missing data for platelets: n = 47.
[e] Missing data for albumin: n = 52.
[f] Missing data for LDH: n = 53.
[g] Missing data for creatinine: n = 48.
[h] Missing data for total bilirubin: n = 59.
[i] Missing data for AST: n = 48.
[j] Missing data for CRP: n = 85.

© 2023 Uehara Y et al. *JAMA Network Open.*

Abbreviations: LDH, lactate dehydrogenase; AST, aspartate transaminase; ALT, alanine transaminase; CRP, C-reactive protein; ULN, upper normal limit; IQR, interquartile range.

© 2023 Uehara Y et al. *JAMA Network Open.*

eTable 3. Characteristics of Patients Participating in Genotype-Matched and Genotype-Nonmatched Trials (Tumor Type and Laboratory Values, n = 241)

**Characteristics**	**All-cancer clinical trials (n = 241)**	**Genotype-matched trials (n = 127)**	**Genotype-non-matched trials (n = 114)**	***P***
Tumor type, n (%)				
Gastrointestinal	86 (36)	44 (35)	42 (37)	.72
Hepatobiliary	56 (23)	30 (24)	26 (23)	
Gynecologic	40 (17)	22 (17)	18 (16)	
Lung	18 (7)	9 (7)	9 (8)	
Sarcoma	11 (5)	8 (6)	3 (3)	
Genitourinary	10 (4)	4 (3)	6 (5)	
Breast	9 (4)	4 (3)	5 (4)	
Other[a]	11 (5)	6 (4)	5 (4)	
Neutrophils (/µL), n (%)				
<1,500	4 (2)	3 (2)	1 (1)	.69
≥1,500	237 (98)	124 (98)	113 (99)	
Hemoglobin (g/dL), n (%)				
<9	4 (2)	3 (2)	1 (1)	.69
≥9	237 (98)	124 (98)	113 (99)	
Platelets ($\times 10^3$/µL), n (%)				
<100	4 (2)	3 (2)	1 (1)	.69
≥100	237 (98)	124 (98)	113 (99)	
Albumin (g/dL), n (%)				
<3.5	19 (8)	11 (9)	8 (7)	.82
≥3.5	222 (92)	116 (91)	106 (93)	
LDH (U/L), n (%)				
<2.5 × ULN	236 (98)	125 (98)	111 (97)	.90
≥2.5 × ULN	5 (2)	2 (2)	3 (3)	
Creatinine (mg/dL), n (%)				
<1.5	240 (99)	126 (99)	114 (100)	>.99
≥1.5	1 (1)	1 (1)	0 (0)	
Total bilirubin (mg/dL), n (%)				
<1.5 × ULN	240 (99)	126 (99)	114 (100)	>.99
≥1.5 × ULN	1 (1)	1 (1)	0 (0)	
AST (U/L), n (%)				
<3.0 × ULN	240 (99)	126 (99)	114 (100)	>.99
≥3.0 × ULN	1 (1)	1 (1)	0 (0)	
CRP (mg/dL)[b], n (%)				

© 2023 Uehara Y et al. *JAMA Network Open.*

Characteristics	All-cancer clinical trials (n = 241)	Genotype-matched trials (n = 127)	Genotype-non-matched trials (n = 114)	*P*
<5	235 (98)	123 (97)	112 (100)	.29
≥5	3 (1)	3 (2)	0 (0)	

[a] Others: head and neck cancers (4), thyroid cancer (2), mesothelioma (1), neuroendocrine tumor (1), melanoma (1), central nervous system tumor (1), and carcinoma of unknown primary (1).

[b] Missing data for CRP: n = 3.
Abbreviations: min, minute; LDH, lactate dehydrogenase; AST, aspartate transaminase; ALT, alanine transaminase; CRP, C-reactive protein; ULN, upper normal limit; IQR, interquartile range.

© 2023 Uehara Y et al. *JAMA Network Open.*

eTable 4. Univariable and Multivariable Analyses of Factors Associated With All–Cancer Clinical Trial Participation by Travel Time to the Hospital (n = 1,127)

	**Univariable analysis**			**Multivariable analysis[a]**		
	**OR**	**95% CI**	***P***	**OR**	**95% CI**	***P***
Travel time ≥ 120 min (vs. < 120 min)	0.68	0.47–0.96	**.034**	0.70	0.47–1.04	.08
Age ≥ 60 years (vs. < 60 years)	0.81	0.61–1.08	.15	0.71	0.51–0.98	**.04**
Gender, women (vs. men)	0.89	0.67–1.19	.43	NA	NA	NA
Performance status ≥ 1 (vs. 0)	0.32	0.24–0.42	**<.001**	0.43	0.31–0.58	**<.001**
Body mass index < 18.5 (vs. ≥ 18.5)	0.71	0.47–1.04	.90	0.99	0.63–1.54	.98
Tumor type, non-common cancers (vs. common cancers)	0.71	0.43–1.15	.18	0.59	0.33–1.03	.07
Number of lines of prior therapies ≥ 2 (vs. < 2)	1.68	1.07–2.67	**.023**	1.91	1.17–3.23	**.01**
Number of metastatic sites ≥ 2 (vs. < 2)	0.71	0.52–0.95	**.022**	0.69	0.48–0.99	**.05**
Liver metastases, yes (vs. no)	0.78	0.59–1.06	.11	0.55	0.38–0.79	**.001**
Brain metastases, yes (vs. no)	0.51	0.22–1.03	.08	0.44	0.18–1.02	.05
Pleural effusions or ascites, yes (vs. no)	0.25	0.13–0.45	**<.001**	0.30	0.15–0.55	**<.001**
Biopsiable, no (vs. yes)	0.43	0.29–0.62	**<.001**	0.27	0.17–0.42	**<.001**
Neutrophils (/µL) < 1,500 (vs. ≥ 1,500)	0.26	0.08–0.63	**.009**	0.24	0.07–0.60	**.007**
Hemoglobin (g/dL) < 9 (vs. ≥ 9)	0.15	0.05–0.37	**<.001**	0.36	0.09–1.01	.06
Platelets (×10^3/µL) < 100 (vs. ≥ 100)	0.28	0.08–0.69	**.015**	0.27	0.08–0.71	**.02**
Albumin (g/dL) < 3.5 (vs. ≥ 3.5)	0.21	0.13–0.34	**<.001**	0.38	0.21–0.64	**<.001**
LDH (U/L) ≥ 2.5 × ULN (vs. < 2.5 × ULN)	0.18	0.06–0.41	**<.001**	0.36	0.12–0.87	**.04**
Creatinine (mg/dL) ≥ 1.5 (vs. < 1.5)	0.17	0.01–1.20	.19	0.47	0.02–2.87	.51
Total bilirubin (mg/dL) ≥ 1.5 × ULN (vs. < 1.5 × ULN)	0.47	0.02–2.52	.47	NA	NA	NA
AST (U/L) ≥ 3.0 × ULN (vs. < 3.0 × ULN)	0.12	0.006–0.54	**.034**	0.14	0.007–0.70	**.05**
CRP (mg/dL) ≥ 5 (vs. < 5)	0.09	0.02–0.25	**<.001**	0.21	0.05–0.62	**.01**
Place of residence, suburban/rural (vs. urban)	1.01	0.73–1.41	.96	NA	NA	NA
Referring hospital, community (vs hospital university hospital or cancer center)	0.89	0.67–1.18	.42	NA	NA	NA
Median income (by zip code) < 25,000$ (vs. ≥ 25,000$)	1.03	0.65–1.60	.89	NA	NA	NA

Note: All variables with *P* < 0.20 were entered into the full model analysis.
Abbreviations: min, minute; LDH, lactate dehydrogenase; AST, aspartate transaminase; ALT, alanine transaminase; CRP, C-reactive protein; ULN, upper normal limit; OR, odds ratio; CI, confidence interval.

© 2023 Uehara Y et al. *JAMA Network Open.*

eTable 5. Univariable and Multivariable Analyses of Factors Associated With All–Cancer Clinical Trial Participation by Travel Distance to the Hospital (n = 1,127)

	Univariable analysis			Multivariable analysis[a]		
	**OR**	**95% CI**	***P***	**OR**	**95% CI**	***P***
Travel distance ≥ 100 km (vs. < 100 km)	0.74	0.52–1.02	.07	0.76	0.51–1.10	.15
Age ≥ 60 years (vs. < 60 years)	0.81	0.61–1.08	.15	0.71	0.51–0.98	**.04**
Gender, women (vs. men)	0.89	0.67–1.19	.43	NA	NA	NA
Performance status ≥ 1 (vs. 0)	0.32	0.24–0.42	**<.001**	0.42	0.31–0.59	**<.001**
Body mass index < 18.5 (vs. ≥ 18.5)	0.71	0.47–1.04	.90	0.99	0.63–1.55	.98
Tumor type, non-common cancers (vs. common cancers)	0.71	0.43–1.15	.18	0.59	0.33–1.03	.07
Number of lines of prior therapies ≥ 2 (vs. < 2)	1.68	1.07–2.67	**.02**	1.89	1.15–3.18	**.01**
Number of metastatic sites ≥ 2 (vs. < 2)	0.71	0.52–0.95	**.02**	0.68	0.48–0.99	**.05**
Liver metastases, yes (vs. no)	0.78	0.59–1.06	.11	0.55	0.38–0.78	**.001**
Brain metastases, yes (vs. no)	0.51	0.22–1.03	.08	0.44	0.18–1.01	.05
Pleural effusions or ascites, yes (vs. no)	0.25	0.13–0.45	**<.001**	0.30	0.15–0.55	**<.001**
Biopsiable, no (vs. yes)	0.43	0.29–0.62	**<.001**	0.27	0.17–0.42	**<.001**
Neutrophils (/µL) < 1,500 (vs. ≥ 1,500)	0.26	0.08–0.63	**.009**	0.24	0.070–0.60	**.007**
Hemoglobin (g/dL) < 9 (vs. ≥ 9)	0.15	0.05–0.37	**<.001**	0.36	0.10–1.02	.06
Platelets (×10^3/µL) < 100 (vs. ≥ 100)	0.28	0.08–0.69	**.02**	0.27	0.077–0.72	**.02**
Albumin (g/dL) < 3.5 (vs. ≥ 3.5)	0.21	0.13–0.34	**<.001**	0.38	0.21–0.63	**<.001**
LDH (U/L) ≥ 2.5 × ULN (vs. < 2.5 × ULN)	0.18	0.06–0.41	**<.001**	0.36	0.12–0.88	**.04**
Creatinine (mg/dL) ≥ 1.5 (vs. < 1.5)	0.17	0.01–1.20	.19	0.47	0.02–2.85	.49
Total bilirubin (mg/dL) ≥ 1.5 × ULN (vs. < 1.5 × ULN)	0.47	0.02–2.52	.47	NA	NA	NA
AST (U/L) ≥ 3.0 × ULN (vs. < 3.0 × ULN)	0.12	0.006–0.54	**.03**	0.13	0.007–0.69	**.05**
CRP (mg/dL) ≥ 5 (vs. < 5)	0.09	0.02–0.25	**<.001**	0.22	0.050–0.63	**.01**
Place of residence, suburban/rural (vs. urban)	1.01	0.73–1.41	.96	NA	NA	NA
Referring hospital, community (vs hospital university hospital or cancer center)	0.89	0.67–1.18	.42	NA	NA	NA
Median income (by zip code) < 25,000$ (vs. ≥25,000$)	1.03	0.65–1.60	.89	NA	NA	NA

Note: All variables with P < 0.20 were entered into the full model analysis.
Abbreviations: LDH, lactate dehydrogenase; AST, aspartate transaminase; ALT, alanine transaminase; CRP, C-reactive protein; ULN, upper normal limit; OR, odds ratio; CI, confidence interval.

© 2023 Uehara Y et al. *JAMA Network Open.*

eFigure 1. Travel Time and Distance Map Centered on the National Cancer Center Hospital

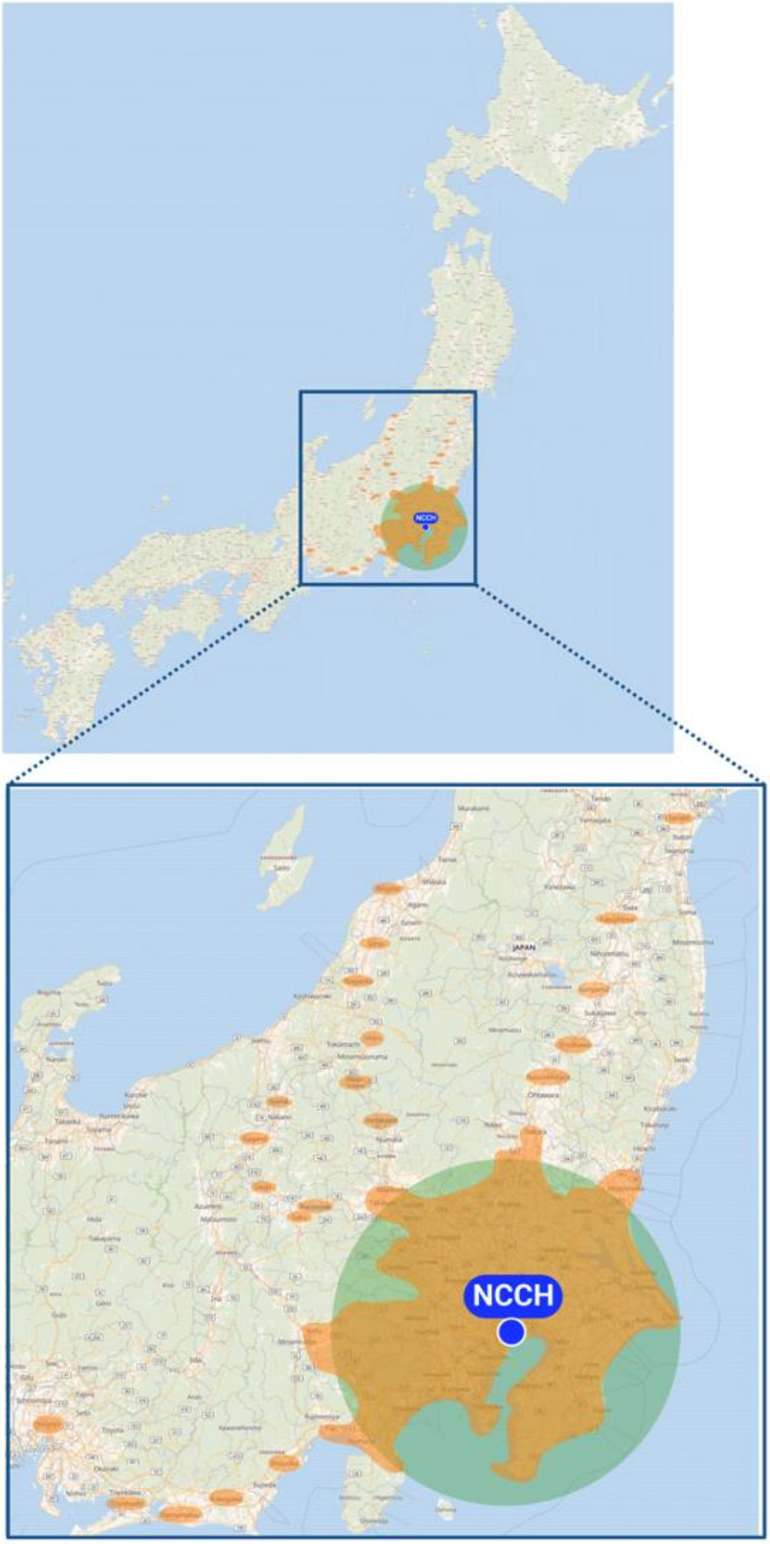

Maps showing the NCCH at the center, marked with a blue circle. The orange area indicates the region within a 120-min travel time from the NCCH. The green circle is mapped within a 100-km radius from the NCCH. The diagram was prepared using Google Maps and BioRender (https://www.biorender.com/).

© 2023 Uehara Y et al. *JAMA Network Open.*

Abbreviations: NCCH, National Cancer Center Hospital.

© 2023 Uehara Y et al. *JAMA Network Open.*

eFigure 2. Correlation Between Travel Time and Distance

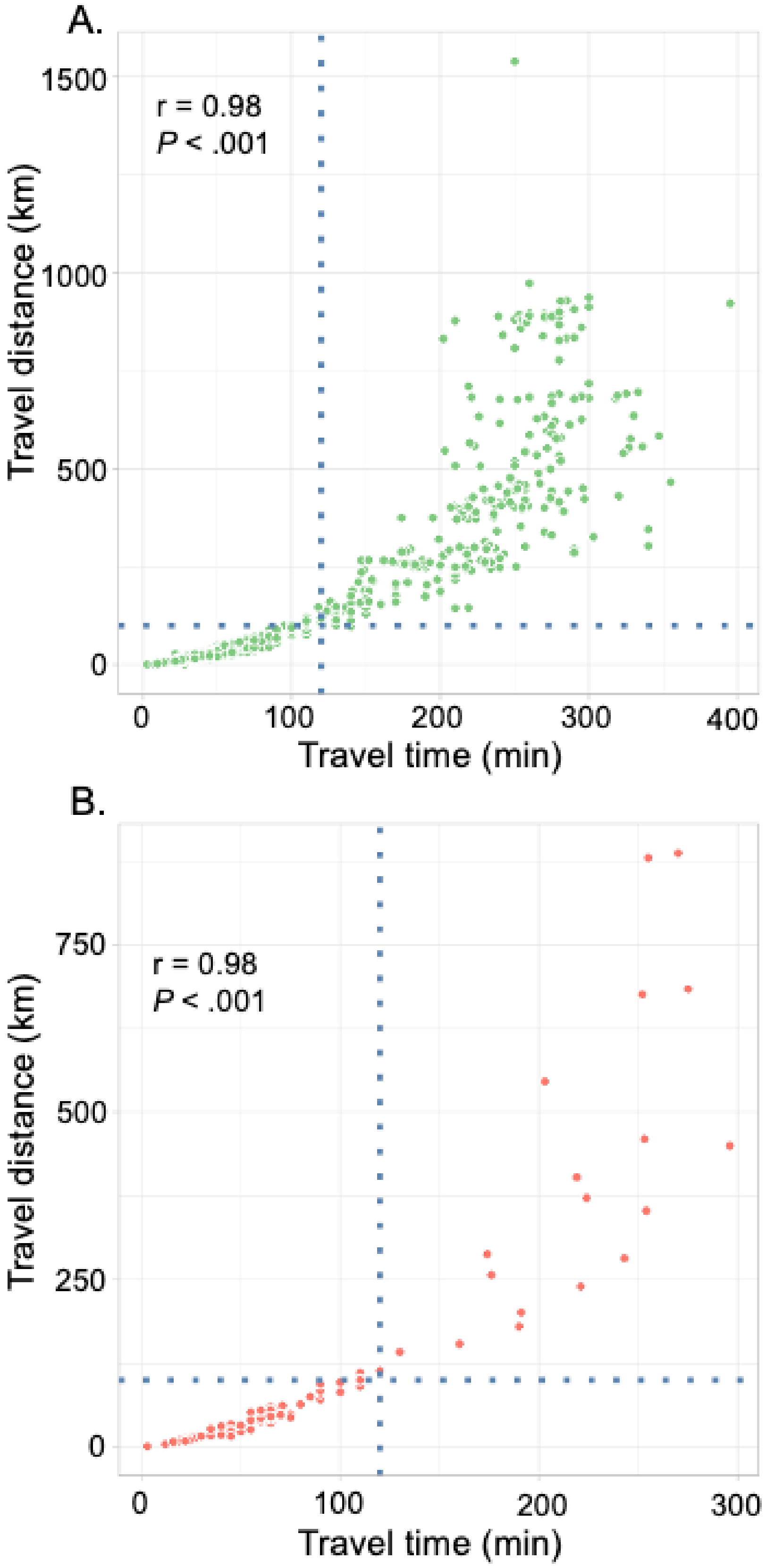

(A) Overall patients (n = 1,127). **(B)** Genotype-matched trial participation (n = 127).

© 2023 Uehara Y et al. *JAMA Network Open.*

eFigure 3. Scatter Plot of Patients With a Travel Time Less Than 120 Minutes and Travel Distance 100 or More km (n = 35)

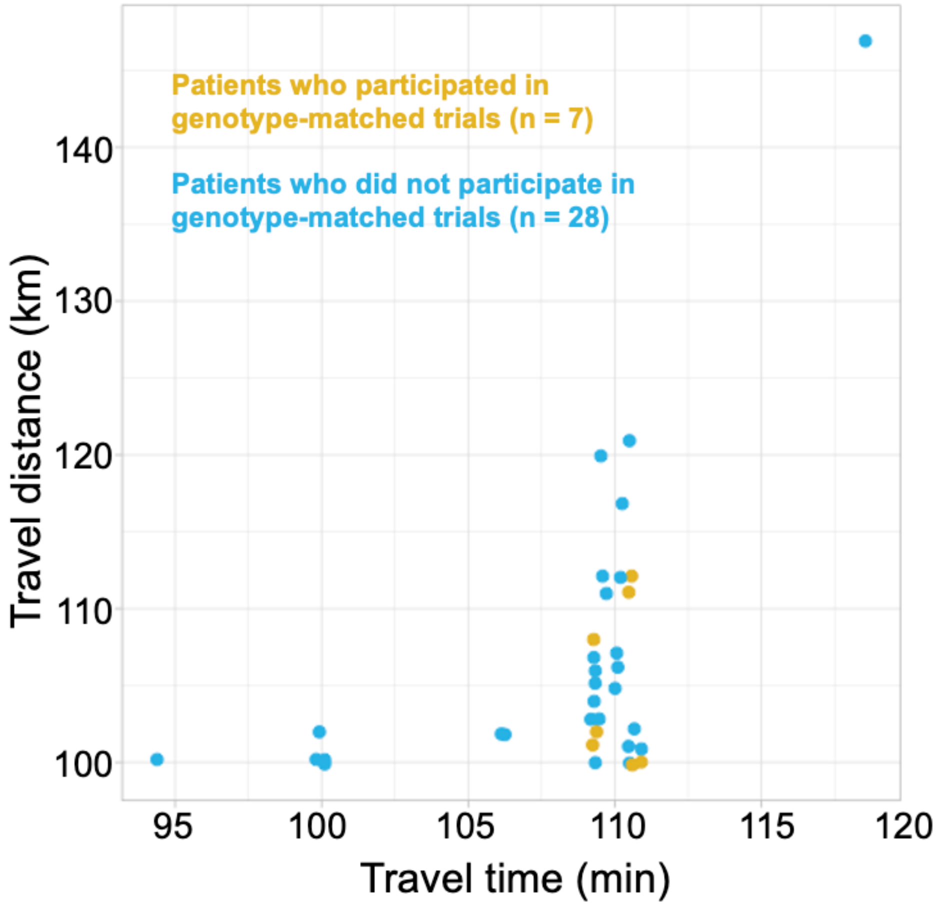

© 2023 Uehara Y et al. *JAMA Network Open.*
