# Supplementary material for: Travel Time and Distance and Participation in Precision Oncology Trials at the National Cancer Center Hospital
Source: JAMA Netw Open. 2023 Sep 15;6(9):e2333188. doi: 10.1001/jamanetworkopen.2023.33188 (PMC10504617; doi:10.1001/jamanetworkopen.2023.33188)
Supplement: Supplement 2. — Data Sharing Statement [file jamanetwopen-e2333188-s002.pdf]

# Data Sharing Statement

Uehara. Travel Time and Distance and Participation in Precision Oncology Trials at the National Cancer Center Hospital. *JAMA Netw Open*. Published September 15, 2023. doi:10.1001/jamanetworkopen.2023.33188

**Data**
**Data available:** No

**Additional Information**
**Explanation for why data not available:** The raw data are not available for public access because of patient privacy concerns but are available from the corresponding author on reasonable request approved by the NCCH institutional review board.
